# Peculiarities of Platelet Metabolism in Patients with Acute Coronary Syndrome with Anxiety–Depressive Disorders and Informativity of Enzymes in the Forecast of Development of Cardiovascular Complications

**DOI:** 10.3390/ph13080169

**Published:** 2020-07-28

**Authors:** Natalya Yu. Shimokhina, Andrey A. Savchenko, Marina M. Petrova

**Affiliations:** 1 Faculty of Medicine, Prof. V. F. Voino-Yasenetsky Krasnoyarsk State Medical University, Partizan Zheleznyak Street 1, 660022 Krasnoyarsk, Russia; aasavchenko@yandex.ru (A.A.S.); stk99@yandex.ru (M.M.P.); 2 Laboratory of Molecular and Cellular Physiology and Pathology, Krasnoyarsk Science Center of the Siberian Branch of the Russian Academy of Sciences, Scientific Research Institute of Medical Problems of the North, Partizan Zheleznyak Street 3g, 660022 Krasnoyarsk, Russia

**Keywords:** acute coronary syndrome, anxiety–depressive disorders, platelets, dehydrogenases, cardiovascular complications

## Abstract

Anxiety–depressive disorders (ADD) are a risk factor of cardiovascular mortality in patients with coronary artery disease (CAD). Acute coronary syndrome (ACS) is the main clinical manifestation of a progressing CAD. Metabolic processes disorder in platelets can be one of the causes of cardiovascular complications in patients with ACS and concomitant ADD. We studied platelets metabolism and prognostic informativity of NAD(P)-dependent dehydrogenases of platelets in ACS patients with ADD in terms of forecasting cardiovascular complications development over a year of observation. The levels of NAD- and NADP-dependent dehydrogenases of platelets were determined by means of a bioluminescent method during the first 24 h after admission to hospital and in dynamics in 10 days. Among 315 examined patients, ADD was found in 161 (51.1%). ACS patients with concomitant ADD had both cytoplasmic and mitochondrial processes impairment in platelets that consisted in a decrease of energy metabolism intensity, inhibition of anaerobic glycolysis reactions and lipid catabolism. After 12 months of follow-up, 41 (25.5%) cardiovascular complications were detected in the group of ACS patients with ADD and 20 (13.0%) in the group of ACS patients without ADD. According to the results of the analysis of the neural network based on NAD(P)-dependent dehydrogenases of platelets activity in ACS patients with ADD, indicators were obtained that are informative for predicting the development of recurrent cardiovascular complications.

## 1. Introduction

According to data found in literature, the prevalence of anxiety–depressive disorders (ADD) in patients with cardiovascular diseases ranges from 20% to 45%, which is 3–4 times higher than among the general population [1,2,3,4]. It is important to note that a risk of cardiovascular mortality in patients with an acute coronary syndrome (ACS) and an ADD was 2–2.6 times higher than for patients without affective disorders [5].

A possible mechanism of a negative interaction between ADD and CAD is a combination of a higher level of catecholamines, a low variability of a cardiac rhythm, an endothelium dysfunction and an activation of inflammation reactions [5,6,7,8].

ACS is the main clinical manifestation of atherosclerosis progression in coronary arteries. Platelets activity and aggregation are key components of an ACS [9,10]. Despite the optimal medical help during the first 30 days of hospitalization after ACS, the risk of unfavorable cardiovascular occurrences is at its highest. However, later on the patients who have had ACS are still subject to a continuous higher risk of recurrent cardiovascular incidents [11,12].

With a high probability a risk of recurrent cardiovascular complications in patients with ACS can be related to functional activity of platelets. A connection between depression and hyperreactivity of platelets has been established [13,14]. Taking into consideration the fact that platelets make a major contribution into the pathogenesis of ACS, the research of metabolic activity of these cells is of a great significance. The synthesis of surface receptors, biologically active substances that are produced by platelets, the condition of cell membranes directly depends on the activity of intracellular processes that in its turn predetermines the reactivity of homeostasis system as a whole including during pathophysiological processes [15,16]. Oxidoreductases have key positions of main metabolic paths. NAD-dependent dehydrogenases catalyze oxidation–reduction reactions of metabolism oxidation paths—glycolysis and Krebs cycle. NADP-dependent dehydrogenases participate in the processes of reduction synthesis, in particular in extra-mitochondrial synthesis of fatty acids and steroids, they are also coenzymes of dehydrogenase pentose phosphate pathway [17]. Thus, the research of these issues will allow one to estimate the intensity of metabolic intracellular processes in platelets.

Guided by the above stated, this research was fulfilled to study the platelets metabolism and to determine the most informative prognostic indices to forecast risks of the development of recurrent cardiovascular incidents in patients with ACS combined with ADD based on the activity of NAD(P)-dependent dehydrogenases of platelets.

## 2. Results

The study comprised 315 ACS patients (150 women and 165 men, their average age is 61.4 ± 0.8). ADD was detected in 161 (51.1%) patients, and in 154 (48.9%) patients no affective spectrum disorders were observed. The patient characteristics are presented in Table 1. There were no statistically significant differences between the groups.

The control group comprised 55 relatively healthy volunteers (28 men and 27 women, their average age was 59.6 ± 1.4).

After the 12-month follow-up there were detected 56 (17.8%) recurrent cardiovascular complications (repeated AMI development, stroke, thromboembolism, post-infarction angina, progressing cardiac deficiency, formation of left ventricle post-infarction aneurysm, etc.). Among ACS patients without ADD, cardiovascular complications were observed in 20 subjects (13.0%) whereas for ACS with ADD group—in 41 subjects (25.5%). The results of prospective observation are summarized in Table 1. As the given data show, 5 patients (3.1%) with ADD and 2 patients (1.3%) without ADD developed a repeated myocardial infarction, 9 patients (5.6%) from the first group and 5 patients (3.2%) from the second group had post-infarction angina. Progressing cardiac deficiency was observed in 5 ACS patients with ADD (3.1%) and in 3 ACS patients without ADD (1.9%). Apart from that, over the follow-up year there were 5 cases (3.1%) of death caused by cardiovascular diseases in the group of ACS patients with ADD, and 1 such case (0.6%) in the group of those without affective disorders.

**Figure 1:**
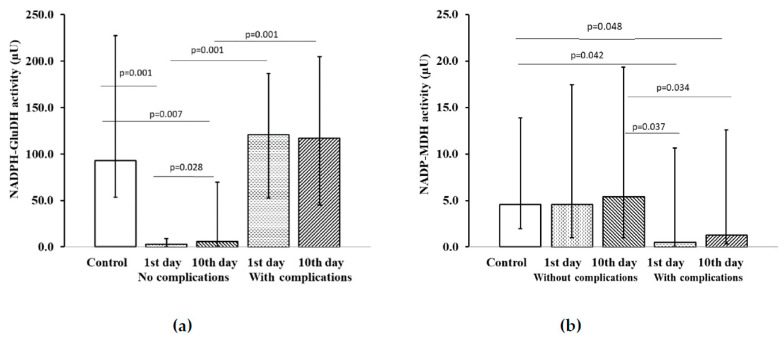

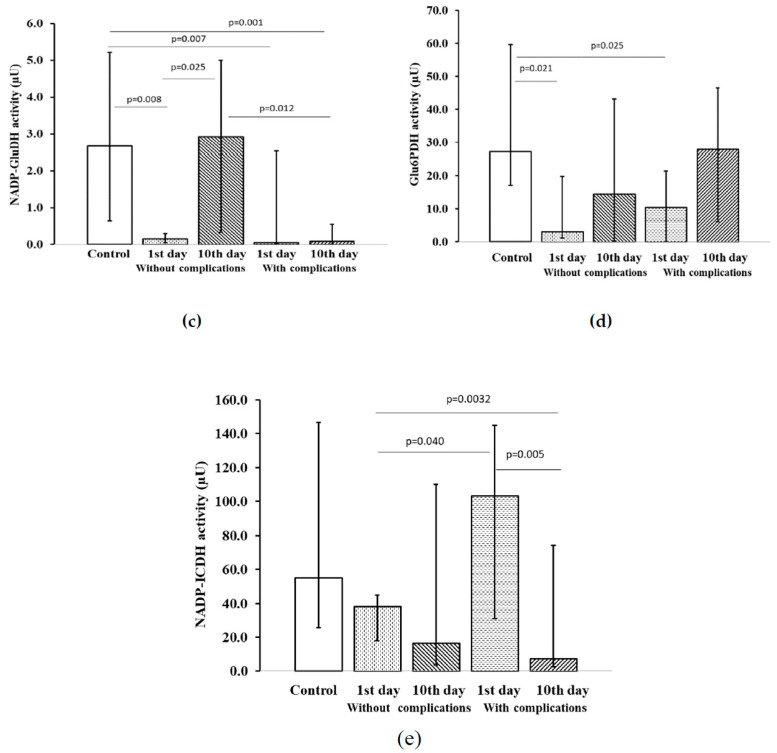
Activity of NADP-dependent platelet dehydrogenases in patients with acute coronary syndrome (ACS) without anxiety–depressive disorders (ADD), depending on the development of cardiovascular complications: (**a**) NADPH–GluDH activity; (**b**) NADP–MDH activity; (**c**) NADP–GluDH activity; (**d**) Glu6PDH activity; (**e**) NADP–ICDH activity. Notes: Data expressed as median and interquartile range (IQR), *p* < 0.05 was considered statistically significant. Wilcoxon matched-pairs test for patients without complications on the 1st vs. 10th day of the examination and for patients with complications on the 1st vs. 10th day of the examination. Mann–Whitney U test for patients vs. control and for patients with complications vs. patients without complications. Notes: µU—microunit.

The study of activity of metabolic enzymes of platelets in ACS patients showed low NADPH–GluDH level on the first day in patients without affective disorders. By the time of discharge from hospital the levels of this enzyme increased to some extent, but still did not reach the control values (Figure 1a). In the examined ACS patients without ADD who developed cardiovascular complications over a year, NADPH–GluDH activity on the first day and on the 10th day of treatment corresponded to the control level, whereas NADP–MDH activity in platelets in ACS patients without affective disorders who had no recurrent cardiovascular complications at all stages of the study did not reliably differ from control values.

In ACS patients with ADD who developed over the follow-up period recurrent cardiovascular complications, there was observed a low NADP–MDH activity on the first day in comparison with both control group data and the results of ACS patients without ADD who had no complications (Figure 1b). By the time of discharge from the hospital on the 10th day, a NADP–MDH activity in ACS patients without ADD, but with emerged recurrent cardiovascular complications increased 6.5 times relative to the initial level, however it remained lower that the control level.

NADP–GluDH activity in patients who had ACS without affective spectrum disorders on the 1st day of the disease was significantly lower than the control numbers regardless of the development of repeated cardiovascular events (Figure 1c). It should be noted that the activity of NADPH–GluDH in platelets remained low in ACS patients without ADD, but with the development of repeated cardiovascular complications on the 10th day of treatment, while in patients without complications, there was an increase to the control level (Figure 1c).

In patients with ACS without affective disorders, there was a decrease in the activity of Glu6PDH on the 1st day of the disease compared to the control group, regardless of the presence or absence of recurrent cardiovascular complications. In addition, regardless of the development of complications, by the time patients were discharged from the hospital, on the 10th day, there was an increase in the Glu6PDH levels in ACS patients without ADD (Figure 1d).

NADP–ICDH activity in ACS patients without ADD, but with recurrent cardiovascular complications during the follow-up period on the 1st day of the disease was 2.7 times higher than the results of patients without recurrent cardiovascular events (Figure 1e). By the time patients were discharged, there was a significant decrease in NADP–ICDH compared to both the 1st day of hospitalization and the control level.

**Figure 2:**
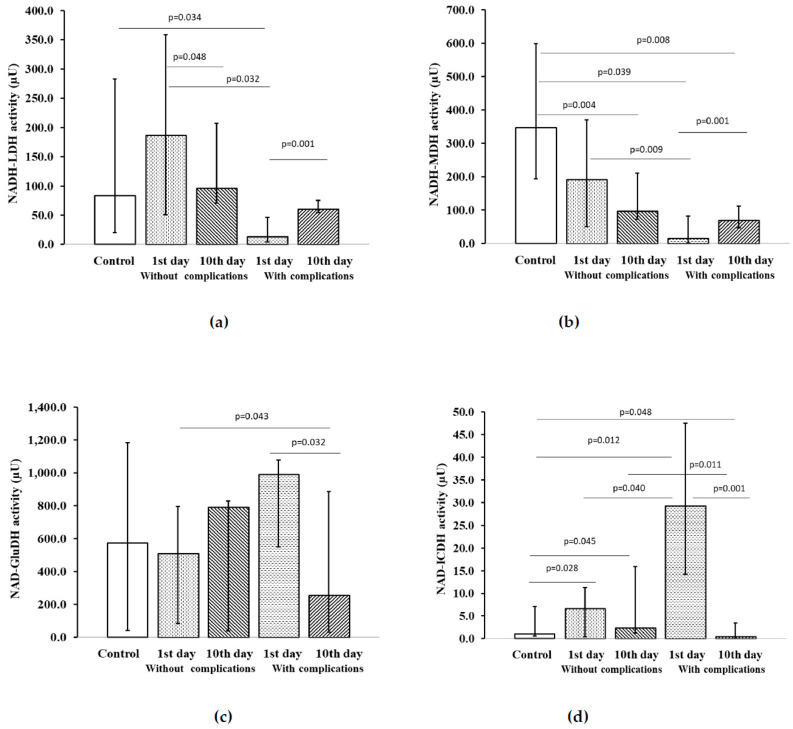
Activity of NAD-dependent platelet dehydrogenases in patients with ACS without ADD, depending on the development of cardiovascular complications: (**a**) NADH–LDH activity; (**b**) NADH–MDH activity; (**c**) NAD–GluDH activity; (**d**) NAD–ICDH activity. Notes: Values expressed as median and interquartile range in the form of 25 and 75 percentiles, *p* < 0.05 was considered statistically significant. Wilcoxon matched-pairs test for patients without complications on the 1st vs. 10th day of the examination and for patients with complications on the 1st vs. 10th day of the examination. Mann–Whitney U test for patients vs. control subjects and for patients with complications vs. patients without complications. Notes: µU—microunit.

Assay of the activity of NAD-dependent platelet dehydrogenases in ACS patients without ADD revealed that on the 1st day of the disease, there was a decrease in NADH–LDH levels in patients with the recurrent cardiovascular events compared to the control results and ACS patients without ADD and cardiovascular complications during the one-year follow-up (Figure 2a).

By the time patients were discharged from the hospital, on the 10th day, the activity of NADH–LDH increased significantly and did not significantly differ from the results of the control group. At the same time, in the group of ACS patients without affective disorders and recurrent cardiovascular events, on the 10th day of observation the level of NADH–LDH decreased by 1.9 times compared to the 1st day of the disease. On the 1st day of hospitalization in ACS patients without ADD, but with recurrent cardiovascular complications, there was a low activity of NADH–MDH in platelets. On the 10th day of follow-up, the levels of this enzyme increased, but still remained below the indicators of the control group (Figure 2b).

Meanwhile, ACS patients without affective disorders and repeated cardiovascular events on the 10th day of treatment had low values of NADH–MDH comparing to the control group. On the 1st day of hospitalization, the activity of NAD–GluDH in ACS patients without ADD, but with recurrent cardiovascular complications, was increased comparing to the patients without such complications (Figure 2c).

By the time of discharge, on the 10th day of treatment, the activity of NAD–GluDH in patients with ACS without affective spectrum disorders, but with recurrent cardiovascular events, decreased by 3.9 times compared to the results of the first day of hospitalization.

In ACS patients without ADD and cardiovascular complications during the follow-up period, the activity of NAD–ICDH on the 1st day of the study was significantly increased comparing to the control group and remained at a high level till the discharge of patients on the 10th day of treatment (Figure 2d). On the 1st day of the disease the NAD–ICDH levels in ACS patients without ADD, but with the development of adverse cardiovascular events during the one-year follow-up were significantly higher than in control group and in the group of ACS patients without ADD and cardiovascular complications. In ACS patients without affective disorders with recurrent cardiovascular events, there was a decrease in the activity of this enzyme on the 10th day of treatment comparing to the control group and results of patients without complications (Figure 2d).

**Figure 3:**
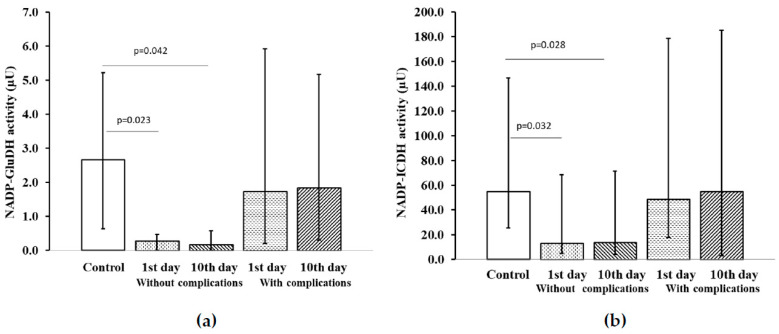

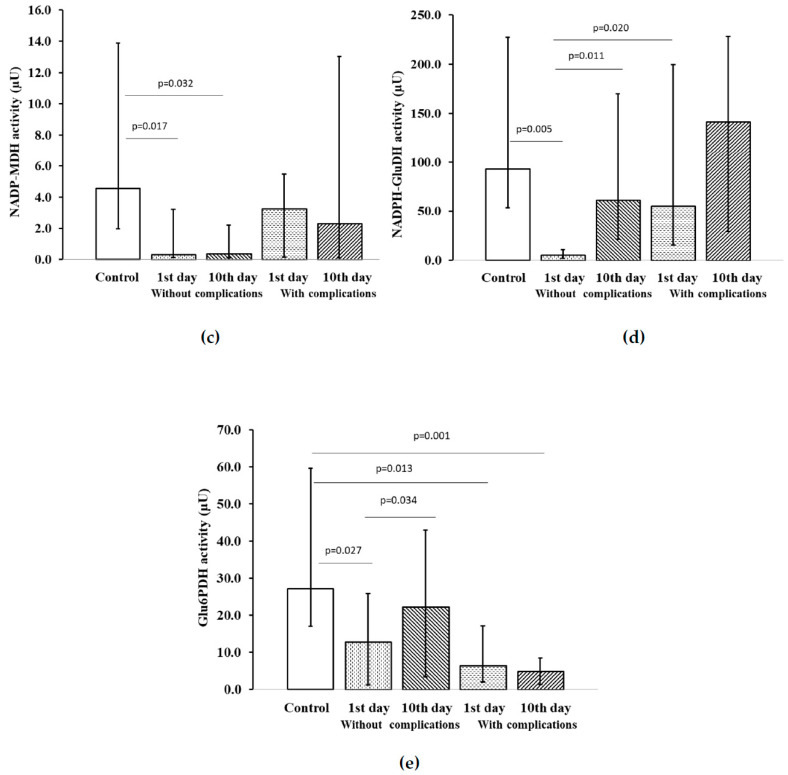
Activity of NADP-dependent platelet dehydrogenases in patients with ACS and ADD, depending on the development of complications: (**a**) NADP–GluDH activity; (**b**) NAD–ICDH activity; (**c**) NADP–MDH activity; (**d**) NADPH–GluDH activity; (**e**) Glu6PDH activity. Notes: Values expressed as median and interquartile range in the form of 25 and 75 percentiles, *p* < 0.05 was considered statistically significant. Wilcoxon matched-pairs rank test for patients without complications on the 1st vs. 10th day of the examination and for patients with complications on the 1st vs. 10th day of the examination. Mann–Whitney U test for patients vs. control subjects and for patients with complications vs. patients without complications. Notes: µU—microunit.

In analysis of the activity levels of NADP-dependent platelet dehydrogenases in ACS patients with ADD without recurrent cardiovascular events on the 1st and 10th days of the study, low levels of NADP–GluDH activity (Figure 3a), NAD–ICDH activity (Figure 3b) and NADP–MDH activity (Figure 3c) were found comparing to the control group.

In ACS patients without ADD and cardiovascular complications, the activity of NADPH–GluDH (Figure 3d) and Glu6PDH (Figure 3e) was reduced on the 1st day of hospitalization comparing to the control group. By the time patients were discharged from the hospital, on the 10th day, the activity of these enzymes significantly increased and did not differ from the results in control group. In ACS patients with ADD and developed adverse recurrent cardiovascular events, low activity of Glu6PDH was observed at all stages of the research, comparing to the control group (Figure 3e).

**Figure 4:**
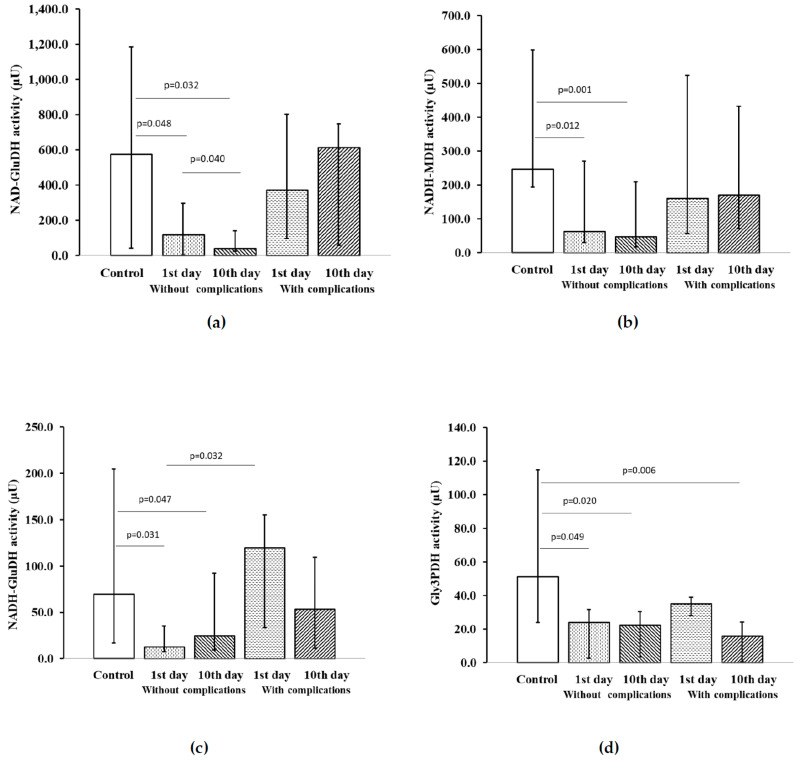

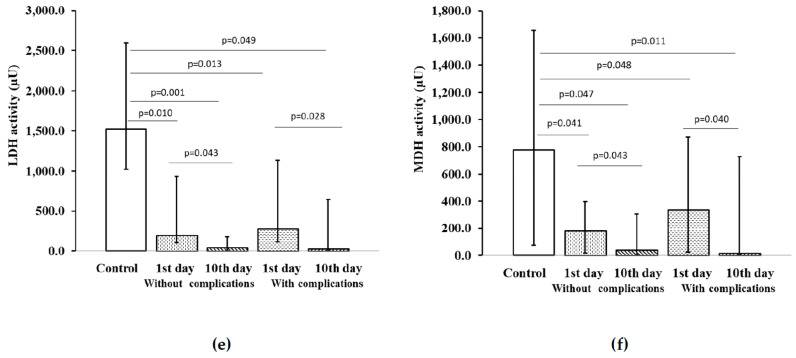
Activity of NAD-dependent platelet dehydrogenases in patients with ACS and ADD, depending on the development of complications: (**a**) NAD–GluDH activity; (**b**) NADH–MDH activity; (**c**) NADH–GluDH activity; (**d**) Gly3PDH activity; (**e**) LDH activity; (**f**) MDH activity. Notes: Values expressed as median and interquartile range in the form of 25 and 75 percentiles, *p* < 0.05 was considered statistically significant. Wilcoxon matched-pairs test for patients without complications on the 1st vs. 10th day of the examination and for patients with complications on the 1st vs. 10th day of the examination. Mann–Whitney U test for patients vs. control subjects and for patients with complications vs. patients without complications. Notes: µU—microunit.

In ACS patients with ADD without repeated cardiovascular events during the one-year follow-up, both on the first day of hospitalization and by the time of discharge from the hospital on the 10th day, low levels of NADH–GluDH activity (Figure 4a), NADH–MDH (Figure 4b) and NADH–GluDH (Figure 4c) were found, compared to the control group. In addition, patients with ACS in combination with affective spectrum disorders, but without cardiovascular complications at all stages of the research, showed low activity of Gly3PDH in platelets compared to the control group (Figure 4d).

Gly3PDH levels were reduced in comparison with control levels when ACS patients with ADD and recurrent cardiovascular events were discharged. In ACS patients with ADD, regardless of the presence or absence of cardiovascular complications, on the 1st day of the disease, low levels of LDH activity (Figure 4e) and MDH activity (Figure 4f) were found in comparison with the control group. By the time of discharge from the hospital of this group of patients, on the 10th day of treatment, the activity of LDH and MDH remained at a low level.

**Figure 5:**
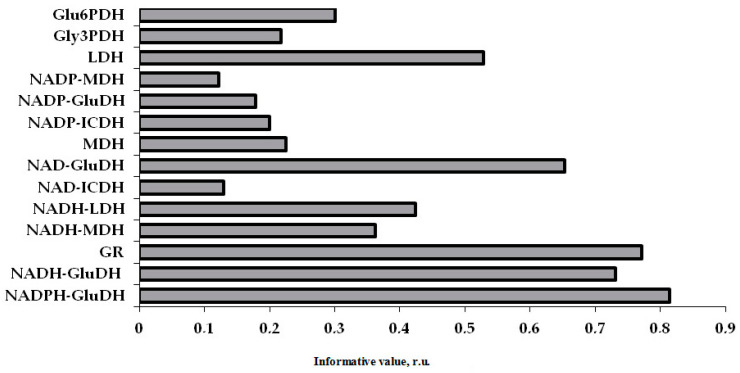
Information content of NAD and NADP-dependent platelet dehydrogenases in the neural network classification in ACS patients with ADD in the prognosis of the development of cardiovascular complications. Notes: r.u.—relative units.

The most informative in the neural network model of classification of patients in predicting the development of repeated adverse cardiovascular events in ACS patients with ADD were the activity levels of NADPH–GluDH, GR and NADH–GluDH (Figure 5).

## 3. Discussion

Platelets play a key role in the hemostatic system [18,19,20]. Disturbances in the blood coagulation system are the main pathogenetic link in the development of ACS, and the presence of concomitant ADD may be one of the reasons for the poor prognosis in this category of patients [21,22,23].

Maintaining the functional activity of platelets largely depends on the state of the processes of cell energy balance. A main source of stable and affordable energy in the blood platelets is mitochondria [24,25,26]. ATP replenishment in platelets is provided by anaerobic glycolysis reactions (substrate phosphorylation) and oxidative phosphorylation processes. In addition, the energy processes in platelets are quite plastic and, in addition to exogenous glucose, as the main metabolic substrate, they are able to use endogenous glycogen and also oxidize fatty acids and amino acids as sources of ATP [15]. As in most cells, the process of utilization of glucose in platelets occurs both under aerobic and anaerobic conditions, and what is more, in the case of platelets activation and the need for rapid generation of ATP, metabolic pathways can switch between oxidative phosphorylation and glycolysis [15,27].

Since platelets activation is an energy-dependent process, for example, secretion, adhesion and aggregation induced by thrombin, collagen or ADP, the study of the dehydrogenase activity will determine the intensity of bioenergetic cell metabolism [15,28]. There is little literature data on the characteristics of bioenergy processes, catabolic and anabolic pathways that occur in blood platelets, both under normal functioning and pathologic conditions.

Analyzing the activity levels of NAD- and NADP-dependent platelets dehydrogenases obtained in our work, we found that only in patients with ACS without ADD with the development of cardiovascular complications in platelets on the 1st and 10 day of treatment, the activity of NADP–MDH was reduced, which allows us to make an assumption about the inhibition of bypass reaction of the Krebs cycle in mitochondria and the reactions of the xenobiotic catabolism [29].

On the 1st day of hospitalization in patients with ACS without ADD and without cardiovascular complications, activity of terminal reactions of platelet glycolysis according to the level of activity of the anaerobic LDH reaction and the NADH-dependent reaction of MDH corresponds to the control range. On the 10th day of treatment, a decrease in the activity of the LDH anaerobic reaction and NADH-dependent reaction of malate dehydrogenase is revealed, which may indicate inhibition of anaerobic glycolysis [30].

At the same time, in patients with ACS without ADD, but with the development of cardiovascular complications during the year of observation, on the 1st day of treatment, a decrease in the activity of the anaerobic LDH reaction and NADH-dependent reaction of malate dehydrogenase which reflects the inhibition of anaerobic glycolysis while on the 10th day of treatment, an increase in the activity of NADH-dependent reaction of lactate dehydrogenase and NADH-dependent reaction of malate dehydrogenase was found. NADH–MDH provides metabolic support of the respiratory chain in mitochondria, ensuring functioning of the malate aspartate shuttle [31].

It can be assumed that the intensity of the substrate flow at terminal reactions of glycolysis will affect the entry of pyruvate into the tricarboxylic acid cycle and accordingly the state of mitochondria aerobic respiration. Indeed, on the 1st day of follow-up, inhibition of NADH-dependent reactions of lactate dehydrogenase and malate dehydrogenase in platelets of patients with ACS without ADD lead to an increased activity of NAD–ICDH and NADP–ICDH which was associated with an increase in the intensity of substrate flow at the initial stage of the citric acid cycle. However, on the 10th day of treatment in patients of this group, the activity of NAD–ICDH and NADP–ICDH decreased. However, NADP–ICDH is known to perform auxiliary dehydrogenase reactions of the mitochondrial compartment, the function of which is aimed at increasing intensity of the substrate flow in the Krebs cycle [32].

Another mechanism for regulating intensity of the substrate flow in the Krebs cycle is the state of activity of NAD- and NADP-dependent glutamate dehydrogenases. On the 1st and 10th days of examination a marked decrease in the activity of NADPH–GluDH, an enzyme that ensures the entry of amino acid metabolism substrates into the Krebs cycle is revealed in ACS patients without concomitant disorders of the affective spectrum and without cardiovascular complications during the year of observation [33]. In patients with cardiovascular complications, the activity of this enzymatic reaction corresponds to the control range. Therefore, the level of NADPH-dependent outflow of substrates from the tricarboxylic acid cycle to the amino acid exchange reactions in the absence of complications is reduced, which allows maintaining the intensity of the substrate flow in the citric acid cycle. It is noteworthy that a high level of prognostic significance of NADPH–GluDH is confirmed by the result of neural network analysis.

Depending on the development of complications, characteristic features were also found in the inflow of substrates onto the Krebs cycle through glutamate dehydrogenase. Thus, in ACS patients without ADD and cardiovascular complications, the activity of NADP–GluDH on the 1st day of follow-up was significantly reduced whereas on the 10th day of follow-up, by the time of discharge from the hospital, it was increased. In patients with cardiovascular complications, the activity of the enzyme was significantly reduced both on the 1st and on the 10th day of treatment.

Meanwhile, on the 1st day of follow-up the activity of NAD–GluDH in patients with complications exceeded that of patients without complications. However, by the 10th day of treatment the activity of the enzyme decreased. Such a state of activity in ACS patients without ADD is characteristic for a low level of NADP-dependent inflow of substrates to the Krebs cycle, regardless of the development of future cardiovascular complications. In ACS patients without cardiovascular complications, as a result of the treatment, the inflow of substrates through NADP–GluDH was restored, while in patients with complications it remained at a low level. At the same time, the level of NAD-dependent inflow of substrates on the energy processes in platelet mitochondria during the development of subsequent cardiovascular complications was higher than in their absence.

There is also another mechanism of metabolic regulation of platelet functions. In recent years, the metabolic mechanisms of epigenetic regulation were actively discussed. It is established that various intracellular metabolites affect epigenetic processes, being substrates or coenzymes of epigenetically modifying enzymes [34,35,36]. NAD^+^, glutamine, α-ketoglutarate, acetyl- coenzyme A and glutathione are identified as the most important metabolites that affect the activity of enzymes that change the structure of chromatin and, consequently, gene expression and cell activity [36,37,38,39].

Therefore, intracellular enzymes that affect the concentration of these metabolites determine the functional cell activity (both through metabolic regulation mechanisms and mechanisms of epigenetic regulation). These metabolic mechanisms characterize the prognostic significance of NADH–GluDH, NADPH–GluDH and GR, as enzymes that determine the concentration of glutamate, α-ketoglutarate, NAD^+^ and glutathione in platelets and affect the epigenome of cells.

## 4. Materials and Methods

### 4.1. Participants and Procedures

The study comprised patients who received hospital treatment in the cardiologic department of Krasnoyarsk Interdistrict Clinical Hospital of Emergency Medical Care named after N.S. Karpovich, Krasnoyarsk, the Russian Federation from 2011 to 2014. The inclusion criteria were the following: ACS in patients of both sexes aged from 35 to 70 years over the first 24 h of admission to hospital from the onset of the disease as well as signed informed consent. Exclusion criteria: concomitant diabetes mellitus and other severe somatic pathology (neoplasms, chronic obstructive pulmonary disease, chronic renal failure, systemic connective tissue diseases, chronic rheumatic heart disease, acute and chronic infectious diseases); the age of patients younger than 35 years and older than 70 years; thrombolysis at the prehospital or stationary stage; heart failure stage III; cardiogenic shock upon admission to the hospital; lack of informed consent. All subjects gave their informed consent for inclusion before their participation in the study. The study was conducted in accordance with the Declaration of Helsinki, and the protocol was approved by the ethics committee of Professor V.F. Voino-Yasenetsky Krasnoyarsk State Medical University (ethical code 35/2011 of 31 October 2011). The diagnosis of ACS and further on that of unstable angina or acute myocardial infarction (AMI) was given according to the recommendations of the European Society of Cardiology [40].

The study of platelets enzymes activity was conducted in both groups of all patients during the first 24 h after their hospitalization to ICU and in dynamics on the 10th day before they were discharged from hospital. For the first 72 h after transfer from ICU, all the patients underwent a psychometric examination using the hospital anxiety and depression scale (HADS). All patients were divided into two groups: ACS patients with ADD formed the first group and ACS patients without ADD formed the second one. No differences in clinical-anamnestic characteristics between the groups were detected (Table 1). In the control group, there was conducted a test designed to detect ADD and a single examination of platelets enzymes activity. In the control group, no cardiovascular diseases and ADD were detected.

All patients were examined again 12 months after being included into the study with the purpose to detect possible cardiovascular complications, such as acute myocardial infarction (AMI) relapse in hospital, repeated myocardial infarction, stroke development, thromboembolism, onset of unstable post-infarction angina, increase of cardiac deficiency functional class, formation of post-infarction aneurysm of the left ventricle and death from cardiovascular causes.

### 4.2. Platelet Isolation

Blood samples were collected from the antecubital vein in vacutainer tube with 3.8% tri-sodium citrate, the morning and on an empty stomach. Blood was centrifuged for 10 min at 140 g at room temperature to produce platelet-rich plasma. Further platelet isolation was carried out according to the method described in article of Chiang J.Y. et al. (2019) with modifications [41]. Evaluation of the number and purity of the isolated platelets was carried out on a hematological analyzer Sysmex XE-5000 (Sysmex, Inc., Mundelein, IL, USA) by a standard method [42]. The purity of the isolated platelets was 98–100%.

### 4.3. Bioluminescent Analysis

Bioluminescent method was used to determine the levels of activity of NAD- and NADP-dependent dehydrogenases in platelets [43]. The activity of the following enzymes was determined: glucose-6-phosphate dehydrogenase (Glu6FDH), glycerol-3-phosphate dehydrogenase (Gly3PDH), NADP-dependent malate dehydrogenase decarboxylated (NADP–MDH), NAD-dependent reaction (LDH) and NADH-dependent reaction of lactate dehydrogenase (NADH–LDH), malate dehydrogenase, NAD-dependent reaction (MDH) and NADH-dependent reaction of malate dehydrogenase (NADH–MDH), NADP-dependent glutamate dehydrogenase (NADP–GluDH) and NADPH-dependent glutamate dehydrogenase (NADPH–GluDH), NAD-dependent glutamate dehydrogenase (NAD–GluDH) and NADH-dependent reaction of glutamate dehydrogenase (NADH–GluDH), NAD-dependent isocitrate dehydrogenase (NAD–ICDH) and NADP-dependent isocitrate dehydrogenase (NADP–ICDH) and glutathione reductase (GR).

The activity of NAD- and NADP-dependent dehydrogenases was expressed in enzymatic units on 1 mg of protein (1 U = 1 µmol/min) [17].

Protein concentration was determined using Pierce™ modified Lowry protein assay kit (Thermo Fisher Scientific, Waltham, MA, USA).

### 4.4. Statistical Analysis

Descriptive statistics for categorical variables are presented as frequencies and percentages. The description of the sample collection was performed by means of median (Me) and an interquartile range of 25 and 75 percentiles (C_25_ and C_75_). To determine the pattern of variables distribution Kolmogorov–Smirnov criterion was used. When distributions were symmetrical the variables are represented as means and standard deviations. The validity of differences between parameters of independent sample collections was estimated by the Mann–Whitney U test. Statistical significance of differences between the parameters of dependent sample collections was estimated by the Wilcoxon matched-pairs test. The differences were considered significant at *p* < 0.05. The estimation of informativity of the studied parameters was performed by neuroclassification model data mining general classification. Statistical analysis was conducted using the Statistica 8.0 software package (StatSoft, Inc., Tulsa, OK, USA, 2007).

## 5. Conclusions

The results of our work indicate that in ACS patients with ADD, the detected changes in the activity of mitochondrial compartment enzymes even against the background of substrate stimulation of the Krebs cycle, indicate insufficient substrate flow through it, this leading to inhibition of aerobic respiration. The established changes in the energy metabolism of cells may be a pathogenetic basis for impaired functional activity of platelets in the process of hemostasis in ACS patients with ADD.

The activity levels of NADPH-dependent glutamate dehydrogenase (NADPH–GluDH), NADH-dependent reaction of glutamate dehydrogenase (NADH–GluDH) and glutathione reductase (GR) were determined as the most informative indices in the prognosis of recurrent cardiovascular complications using neural network analysis and obtained data on the enzyme activity in platelets of ACS patients with ADD.

## Figures and Tables

**Table 1:** Patient characteristics and results of patient follow-up 12 months after inclusion in the study.

Parameter	Patients with ACS ^(1)^ and ADD ^(2)^ (*n* = 161)	Patients with ACS without ADD (*n* = 154)	*p*-Value
Age, mean ± SD	64.2 ± 0.8	62 ± 1.1	*p* > 0.05 ^ns^
Men, *n* (%)	86 (53.4)	83 (53.9)	*p* > 0.05 ^ns^
Women, *n* (%)	75 (46.6)	71 (46.1)	*p* > 0.05 ^ns^
STEMI ^(3)^, *n* (%)	40 (24.8)	29 (18.8)	*p* > 0.05 ^ns^
NSTEMI ^(4)^, *n* (%)	49 (30.4)	58 (37.7)	*p* > 0.05 ^ns^
Unstable angina, *n* (%)	72 (44.7)	67 (43.2)	*p* > 0.05 ^ns^
Associated conditions, *n* (%)			
Smoking	81 (50.3)	75 (48.7)	*p* > 0.05 ^ns^
Arterial hypertension	148 (91.9)	134 (87.0)	*p* > 0.05 ^ns^
History of MI ^(5)^	46 (28.6)	33 (21.4)	*p* > 0.05 ^ns^
History of stroke/TIA ^(6)^	25 (15.5)	20 (13.0)	*p* > 0.05 ^ns^
History of coronary angioplasty and stenting	22 (13.7)	19 (12.3)	*p* > 0.05 ^ns^
Dyslipidemia	90 (55.9)	83 (53.9)	*p* > 0.05 ^ns^
Use of antiplatelet agents before hospitalization	72 (44.7)	63 (40.9)	*p* > 0.05 ^ns^
Management of ACS in the hospital, *n* (%)			
Aspirin	156 (96.9)	147 (95.5)	*p* > 0.05 ^ns^
Clopidogrel	146 (90.7)	143 (92.9)	*p* > 0.05 ^ns^
Ticagrelor	16 (9.9)	17 (11.0)	*p* > 0.05 ^ns^
Enoxaparin	143 (88.9)	134 (87.0)	*p* > 0.05 ^ns^
Fondaparinux	9 (5.6)	11 (7.1)	*p* > 0.05 ^ns^
Unfractionated heparin	9 (5.6)	9 (5.8)	*p* > 0.05 ^ns^
PCI ^(7)^ with stenting	115 (71.4)	114 (74.0)	*p* > 0.05 ^ns^
Antiplatelet agents after 12 months of follow-up, *n* (%)			
Aspirin	126 (78.2)	132 (85.7)	*p* > 0.05 ^ns^
Clopidogrel	90 (55.9)	89 (57.8)	*p* > 0.05 ^ns^
Ticagrelor	7 (4.3)	6 (3.9)	*p* > 0.05 ^ns^
Cardiovascular complications, *n* (%)			
Relapse AMI ^(8)^	5 (3.1)	3 (1.9)	*p* > 0.05 ^ns^
Repeated AMI	5 (3.1)	2 (1.3)	*p* > 0.05 ^ns^
Stroke	4 (2.5)	3 (1.9)	*p* > 0.05 ^ns^
Post-infarction angina	9 (5.6)	5 (3.2)	*p* > 0.05 ^ns^
Heart failure progression	5 (3.1)	3 (1.9)	*p* > 0.05 ^ns^
Thromboembolism	4 (2.5)	1 (0.6)	*p* > 0.05 ^ns^
Postinfarction left ventricular aneurysm	4 (2.5)	2 (1.3)	*p* > 0.05 ^ns^
Fatal outcome	5 (3.1)	1 (0.6)	*p* > 0.05 ^ns^
Total complications	41 (25.5)	20 (13.0)	*p* > 0.05 ^ns^

^(1)^ acute coronary syndrome; ^(2)^ anxiety depressive disorders; ^(3)^ ST-elevation myocardial infarction; ^(4)^ non-ST-elevation myocardial infarction; ^(5)^ myocardial infarction; ^(6)^ transient ischemic attack; ^(7)^ percutaneous coronary intervention; ^(8)^ acute myocardial infarction. Data are represented as number of patients (*n*),% and as mean and standard deviation (SD). Mann–Whitney U test for patients with ADD vs. patients without ADD; ns—not significant.
